# Glutamatergic regulation of ghrelin-induced activation of the mesolimbic dopamine system

**DOI:** 10.1111/j.1369-1600.2010.00231.x

**Published:** 2011-01

**Authors:** Elisabet Jerlhag, Emil Egecioglu, Suzanne L Dickson, Jörgen A Engel

**Affiliations:** 1 Section for Pharmacology, Institute of Neuroscience and Physiology, The Sahlgrenska Academy at the University of GothenburgGothenburg, Sweden; 2 Section for Physiology/Endocrinology, Institute of Neuroscience and Physiology, The Sahlgrenska Academy at the University of GothenburgGothenburg, Sweden

**Keywords:** Dopamine, ghrelin, glutamate, opioid, orexin, reward

## Abstract

Recently, we demonstrated that the central ghrelin signalling system, involving the ghrelin receptor (GHS-R1A), is important for alcohol reinforcement. Ghrelin targets a key mesolimbic circuit involved in natural as well as drug-induced reinforcement, that includes a dopamine projection from the ventral tegmental area (VTA) to the nucleus accumbens. The aim of the present study was to determine whether it is possible to suppress ghrelin's effects on this mesolimbic dopaminergic pathway can be suppressed, by interrupting afferent inputs to the VTA dopaminergic cells, as shown previously for cholinergic afferents. Thus, the effects of pharmacological suppression of glutamatergic, orexin A and opioid neurotransmitter systems on ghrelin-induced activation of the mesolimbic dopamine system were investigated. We found in the present study that ghrelin-induced locomotor stimulation was attenuated by VTA administration of the N-methyl-D-aspartic acid receptor antagonist (AP5) but not by VTA administration of an orexin A receptor antagonist (SB334867) or by peripheral administration of an opioid receptor antagonist (naltrexone). Intra-VTA administration of AP5 also suppressed the ghrelin-induced dopamine release in the nucleus accumbens. Finally the effects of peripheral ghrelin on locomotor stimulation and accumbal dopamine release were blocked by intra-VTA administration of a GHS-R1A antagonist (BIM28163), indicating that GHS-R1A signalling within the VTA is required for the ghrelin-induced activation of the mesolimbic dopamine system. Given the clinical knowledge that hyperghrelinemia is associated with addictive behaviours (such as compulsive overeating and alcohol use disorder) our finding highlights a potential therapeutic strategy involving glutamatergic control of ghrelin action at the level of the mesolimbic dopamine system.

## INTRODUCTION

Ghrelin, a gastric hormone, exerts orexigenic and pro-obesity effects by interacting with key brain circuits involved in appetite and energy balance (Tschöp, Smiley & Heiman 2000; Wren *et al*. 2000; Cummings *et al*. 2001; Nakazato *et al*. 2001). The cloned receptor for ghrelin (GHS-R1A) is present, however, not only in discrete hypothalamic cell groups regulating energy balance but also in a number of other central nervous system (CNS) sites, such as the hippocampus, brainstem and reinforcement areas (Guan *et al*. 1997) implying a role for ghrelin in brain reinforcement. Thus, ghrelin activates a key mesolimbic circuit involved in natural as well as drug-induced reinforcement, the cholinergic-dopaminergic reward link (Jerlhag *et al*. 2006, 2007, 2008). This link encompasses the well-described dopamine projection from the ventral tegmental area (VTA) to the nucleus accumbens (N.Acc.) that forms part of the mesolimbic dopamine system, together with a cholinergic projection from the laterodorsal tegmental area (LDTg) to the VTA. By activating this reinforcement link, ghrelin may increase the incentive value of motivated behaviours such as food and drug seeking (Abizaid *et al*. 2006; Jerlhag *et al*. 2006; Jerlhag *et al*. 2009). Indeed, alcohol reinforcement was absent in pharmacological and genetic models of suppressed ghrelin signalling (Jerlhag *et al*. 2009).

Peripherally injected ghrelin also stimulates the mesolimic dopamine system (Jerlhag 2008; Quarta *et al*. 2009), hypothesizing that peripherally produced ghrelin reaches deeper brain structures. Moreover, an important premise for the study is that the afferents regulate ghrelin's effects on the mesolimbic dopamine system involving GHS-R1A in VTA. In the present experiments therefore we sought to determine whether VTA administration of a GHS-R1A antagonist suppresses the locomotor stimulatory and accumbal dopamine releasing effects of peripheral ghrelin. The activity of VTA dopamine neurons is modulated by various afferents to the VTA including glutamate, opioids and orexin (Kalivas, Churchill & Klitenick 1993; Wise 2002). Previously, a role for NMDA, opioid as well as orexin A receptors in different aspects of ghrelin-induced activation of the mesolimbic dopamine systems has been suggested (Toshinai *et al*. 2003; Naleid *et al*. 2005; Abizaid *et al*. 2006). Inspired by the possibility to suppress natural and chemical drug reinforcement by agents that interrupt central ghrelin signalling, we also sought to determine whether ghrelin's ability to activate the mesolimbic dopamine system, as measured by locomotor stimulation and accumbal dopamine release, can be interrupted by pharmacological suppression of glutamatergic, opioid and orexin A systems.

## MATERIALS AND METHODS

### Animals

Adult post-pubertal age-matched male NMRI mice (8–12 weeks old and 25-30 g body weight; B&K Universal AB, Sollentuna, Sweden) were used for studies of locomotor activity and dopamine release as such studies are well-documented in this strain (Jerlhag *et al*. 2006; Jerlhag *et al*. 2007; Jerlhag *et al*. 2008). Upon arrival the mice were allowed to habituate in groups of eight in standard cages (Macrolon III: 400 × 250 × 150 mm), for at least one week before initiation of the experiment. All mice were maintained at 20°C with 50% humidity and a 12/12 hour light/dark cycle (lights on at 7 am). Tap water and food (Normal chow; Harlan Teklad, Norfolk, England) were supplied *ad libitum*, except during the experiments. All experiments were conducted during the day time when the mice are less active. Studies were approved by the Ethics Committee for Animal Experiments in Gothenburg, Sweden.

### Drug administration

Acylated rat ghrelin (Bionuclear; Bromma, Sweden) was diluted in 0.9% sodium chloride (saline vehicle) and was administrated intraperitoneally (i.p.) (10 ml/kg body weight). The selected dose, 0.33 mg/kg, was determined previously, as it increases locomotor activity and accumbal dopamine release as well as induces a conditioned place preference in mice (Jerlhag 2008). Ghrelin was administered 10 minutes prior to the initiation of the experiment (locomotor activity or microdialysis).

The dose of BIM28163 (Ipsen Biomeasure Inc, Milford, MA, USA), a GHS-R1A antagonist, has also been determined previously (Halem *et al*. 2004; Jerlhag *et al*. 2009). BIM28163 was diluted in Ringer solution (NaCl 140 mM; CaCl_2_ 1.2 mM; KCl 3.0 mM and MgCl_2_ 1.0 mM) (Merck KgaA, Darmstadt, Germany) and was administered at a dose of 2.5 µg/side (uni- or bilaterally into the VTA) at 40 minutes prior to i.p. ghrelin/vehicle exposure. Previous studies have established that this compound is a GHS-R1A antagonist and fully inhibits ghrelin-induced GHS-R1A activation (Halem *et al*. 2004).

**Figure 1:**
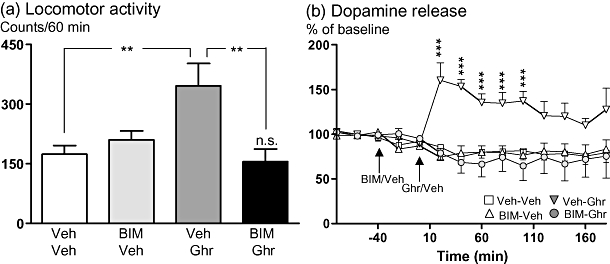
The ghrelin-induced locomotor stimulation and increased accumbal dopamine release are abolished by VTA treatment of the GHS-R1A antagonist BIM28163. (a) Ghrelin (0.33 mg/kg)-induced locomotor stimulation was attenuated by VTA administration of BIM28163 (2.5 µg/side) to but not by vehicle injection in mice (F(3,25) = 5.45, *P* = 0.005) (*n* = 6–8; **P < 0.01; n.s. *P* > 0.05 for Veh-Veh vs. BIM-Ghrelin, Tukey's HSD post-hoc test). (b) We first demonstrated a significant effect of systemic ghrelin to increase dopamine release in comparison to vehicle treatment (*P* = 0.003) and secondly we showed that pre-treatment with BIM28163 (into the VTA) attenuated the ghrelin-induced increase in dopamine release compared to vehicle pre-treatment (*P* = 0.001) (treatment F(3,26) = 6.39, *P* = 0.002; time F(13,338) = 1.77, *P* = 0.047; treatment-time interaction F(13,338) = 4.01, *P* < 0.001). This difference was evident at the time intervals 20–100 minutes (*n* = 7–8; ***P < 0.001, Tukey's HSD post-hoc test).

The selected dose of AP5 (Sigma-Aldrich, Stockholm, Sweden), an N-methyl-D-aspartic acid (NMDA) receptor antagonist, was determined in a dose-response study where 0.5 µg/side (uni-or bilaterally into the VTA) was the highest dose not to affect locomotor activity *per se* (Fig. 1). AP5 or Ringer vehicle were administered 10 minutes prior to i.p. ghrelin/vehicle administration. AP5 does not affect nicotinic acetylcholine receptors in the CNS (Davies & Watkins 1982).

The selected dose of SB334867 (Tocris, Bristol, United Kingdom), an orexin A receptor antagonist, was determined in a dose-response study where 5 µg/side (bilaterally into the VTA) was the highest dose not to affect locomotor activity *per se* (data not shown). Doses in a similar range have previously been shown to block the cue-induced reinstatement of cocaine seeking (Smith, See & Aston-Jones 2009). SB334867 or vehicle (10%-DMSO in Ringer vehicle; Merck KgaA) were administered 10 minutes prior to i.p. ghrelin/vehicle exposure.

Naltrexone, an unselective opioid receptor antagonist with some selectivity to the µ receptor, was diluted in saline vehicle. Naltrexone (1 mg/kg, i.p.) or saline vehicle were injected 30 minutes prior to i.p. ghrelin/vehicle. The dose was determined from previous studies in which doses in a similar range have been shown to block the reinforcing properties of alcohol in rodents (Herz 1997). The rationale for administering by the i.p. route is that direct mesolimbic effects of nalrexone to interrupt ghrelin-induced reinforcement are unlikely, based on previous studies in which this antagonist had no effect on ghrelin-induced food intake when administered into discrete mesolimbic sites (Naleid *et al*. 2005).

Intra-VTA injections were made using a volume of 0.5 µl/side via chronically implanted catheters, over 60 seconds. A 5 µl syringe (Kloehn, microsyringe; Skandinaviska Genetec AB, V. Frölunda, Sweden) was used to facilitate drug administration into the VTA. After each injection, the cannula was left in place for a further 60 seconds to facilitate diffusion. Potentially, the volume administered during intra-VTA injections could raise concerns about specificity due to possible leakage into neighboring structures. Previously, however, we found that only ‘on-target’ placements (in the VTA and LDTg but not in closely adjacent sites) resulted in significant effects of ghrelin or GHS-R1A antagonists on locomotor stimulation and dopamine release using a larger volume (1 µl) for mice (Jerlhag *et al*. 2007, 2009). Supportively, in the present study neither AP5 nor BIM28163 blocked ghrelin-induced locomotor stimulation nor accumbal dopamine release in a few mice in which the canulae were misplaced in neighbouring structures (data not shown). All drug challenges were part of a balanced design with regard to both the treatment order and the number of subjects per treatment.

### Locomotor activity experiments

Peripheral administration of ghrelin has previously been shown to stimulate locomotor activity in mice (Jerlhag 2008). Locomotor stimulation is, at least in part, mediated by an increase in the extracellular concentration of accumbal dopamine (Engel *et al*. 1988). Locomotor stimulation has been suggested to be an homologous effect evolving from a common mechanism involving the mesolimbic dopamine system, implying that locomotor activity reflects reinforcement induced by drugs of abuse (Imperato & Di Chiara 1986; Wise & Bozarth 1987; Engel *et al*. 1988). Thus, accumbal dopamine measurement experiments were conducted only after first establishing that aforementioned antagonist compounds suppress ghrelin-induced locomotor stimulation. All mice, except those treated with naltrexone, were implanted with bilateral guide cannulaes aiming at the VTA. The mice were anesthetized with isofluran (Isofluran Baxter; Univentor 400 Anaesthesia Unit, Univentor Ldt., Zejtun, Malta), placed in a stereotaxic frame (David Kopf Instruments; Tujunga, CA, USA) and kept on a heating pad to prevent hypothermia. The scull bone was exposed and two holes for the guide cannulas (stainless steel, length 10 mm, with an o.d./i.d. of 0.6/0.45 mm) and one for the anchoring screw were drilled. The VTA coordinates were 3.4 posterior to bregma, ±0.5 mm lateral to the midline and 1.0 mm below the brain surface (Franklin & Paxinos 1996). All guide cannulae were surgically implanted four days prior to the experiment. After surgery the mice were kept in individual cages (Macrolon III). At the time of the experiment, a cannula for drug administration was inserted and extended another 3.8 mm ventrally beyond the tip of the guide cannula, aiming at the VTA.

Locomotor activity was registered in eight sound attenuated, ventilated and dim lit locomotor boxes (420 × 420 × 200 mm, Kungsbacka mät- och reglerteknik AB, Fjärås, Sweden). Five by five rows of photocell beams, at the floor level of the box, creating photocell detection allowed a computer-based system to register the activity of the mice. Locomotor activity was defined as the accumulated number of new photocell beams interrupted during a 60-minute period.

Before initiating the experiments, a dummy cannula was carefully inserted and retracted into the guide cannula to remove clotted blood and hamper the spreading depression. Mice were then allowed to habituate to the locomotor activity box one hour prior to drug challenge. In separte experiments, the effects of i.p. administered ghrelin on locomotor stimulation was investigated following intra-VTA administration of BIM28163, AP5 or SB334867 to mice. In subsequent experiments, naltrexone was injected i.p. prior to ghrelin. In the first locomotor activity experiment, BIM28163 (2.5 µg/side) or an equal volume (0.5 µl/side) of vehicle solution (Ringer) was administered locally and bilaterally into the VTA. Ghrelin (0.33 mg/kg) or an equal volume of vehicle solution (saline vehicle) was thereafter injected. The same experimental protocol was used for AP5 (0.5 µg/side), SB334867 (5 µg/side) and naltrexone (1 mg/kg, i.p.). All mice received drug treatment only twice (antagonist/vehicle and ghrelin/vehicle). Neither water nor food was available to the mice during the locomotor experiments. The activity registration started five minutes after the last injection and was subsequently measured for a 60-minute period. For intra-VTA administration only mice with guide cannulae placements in the VTA were included in the statistical analysis.

### In vivo microdialysis and dopamine release measurements

For measurements of extracellular dopamine levels and overflow (that reflect dopamine release), mice were implanted unilaterally with a microdialysis probe positioned in the N.Acc. shell and a guide cannulae into the VTA. The probe and the guide cannula/e were positioned ipsilateral, and the location was randomly alternated to either the left or right side. The surgery was preformed two days prior to the experimental day as described above (see *Locomotor activity experiments*). The coordinates for the N.Acc. shell were: 1.5 mm anterior to the bregma, ± 0.7 lateral to the midline and 4.7 mm below the surface of the brain and the coordinates for the VTA were 3.4 posterior to bregma, ±0.5 mm lateral to the midline and 1.0 mm below the brain surface (Franklin & Paxinos 1996). At the time of the experiment a cannula for drug administration was inserted and extended another 3.8 mm ventrally beyond the tip of the guide cannula, aiming at the VTA. The exposed tip of the dialysis membrane (20 000 kDa cut off with an o.d./i.d. of 310/220 µm, HOSPAL, Gambro, Lund, Sweden) of the probe was 1 mm.

In separate experiments, the effects of intra-VTA administration of AP5, or in separate experiments BIM28163, on ghrelin-induced accumbal dopamine release were determined, involving microdialysis in freely moving mice. On the day of the experiment, a dummy cannula was carefully inserted and retracted into the guide cannula. The probe was thereafter connected to a microperfusion pump (U-864 Syringe Pump; AgnThós AB) and perfused with Ringer solution at a rate of 1.5 µl/minute. After one hour of habituation to the microdialysis set-up, perfusion samples were collected every 20 minutes. The baseline dopamine level was defined as the average of three consecutive samples before the first drug/vehicle challenge. After the baseline samples, the antagonist (AP5 or BIM28163) was administered locally into the VTA followed subsequently by a ghrelin (i.p.) injection. The dopamine levels in the dialysates were determined by HPLC with electrochemical detection. A pump (Gyncotec P580A; Kovalent AB; V. Frölunda, Sweden), an ion exchange column (2.0 × 100 mm, Prodigy 3 µm SA; Skandinaviska GeneTec AB; Kungsbacka, Sweden) and a detector (Antec Decade; Antec Leyden; Zoeterwoude, the Netherlands) equipped with a VT-03 flow cell (Antec Leyden) were used. The mobile phase (pH 5.6), consisting of sulfonic acid 10 mM, citric acid 200 mM, sodium citrate 200 mM, 10% EDTA, 30% MeOH, was vacuum filtered using a 0.2 µm membrane filter (GH Polypro; PALL Gelman Laboratory; Lund, Sweden). The mobile phase was delivered at a flow rate of 0.2 ml/minute passing a degasser (Kovalent AB), and the analyte was oxidized at +0.4 V.

After completion of the microdialysis experiments, the locations of the probe and guide cannulae were verified. Neither water nor food were available to the mice during the microdialysis experiment. Only mice with probe placement in the N.Acc. and guide cannulae in the VTA were included in the statistical analysis.

### Verification of probe and cannula/e placement

After the locomotor activity and microdialysis experiments were completed, the location of the probe and/or cannula/e were verified. The mice were decapitated, probes were perfused with pontamine sky blue 6BX to facilitate probe localization, and the brains were mounted on a vibroslice device (752M Vibroslice; Campden Instruments Ltd, Loughborough, UK). The brains were cut in 50 µm sections and the location of the probe and/or cannula/e was determined by gross observation using light microscopy. The exact position (some correct and some misplaced) of the probe and/or guide cannula/e was verified (Franklin & Paxinos 1996).

### Statistical analyses

All locomotor activity data were evaluated by a two-way ANOVA followed by Tukey's HSD post-hoc tests comparing treatments. The microdialysis experiments were evaluated by a two-way ANOVA for repeated measures followed by Tukey's HSD post-hoc test for comparisons between different treatments and specifically at given time points. Data are presented as mean ± SEM. A probability value of *P* < 0.05 was considered as statistically significant.

## RESULTS

### Effects of intra-VTA administration of a GHS-R1A antagonist on ghrelin-induced locomotor stimulation and accumbal dopamine release in mice

First, the role of GHS-R1A receptors in the VTA for the reinforcing effects of ghrelin by tests of ghrelin-induced locomotor stimulation and, in separate studies, by measurement of ghrelin-induced dopamine release were investigated. The locomotor stimulatory and accumbal dopamine releasing effects of ghrelin were attenuated by local administration of the GHS-R1A antagonist BIM28163 into the VTA (Fig 1a,b), at a dose shown previously to have no effect on locomotor stimulation and accumbal dopamine release *per se* (Jerlhag *et al*. 2009). Thus, ghrelin-induced locomotor stimulation (*P* < 0.01) was attenuated by VTA administration of BIM28163 (*P* < 0.01) in mice (F(3,25) = 5.45, *P* = 0.005: *n* = 6–8). In the microdialysis experiments a significant effect of systemic ghrelin to increase dopamine release in comparison to vehicle treatment was observed (*P* = 0.003). Pre-treatment with BIM28163 attenuated the ghrelin-induced increase in dopamine release compared with vehicle pre-treatment in mice (*P* = 0.001) (treatment F(3,26) = 6.39, *P* = 0.002; time F(13,338) = 1.77, *P* = 0.047; treatment-time interaction F(13,338) = 4.01, *P* < 0.001). This difference was evident at the time intervals 20–100 minutes (*P* < 0.001: *n* = 7–8).

### Effects of intra-VTA administration of an orexin A receptor antagonist or peripheral injection of an opioid receptor antagonist on ghrelin-induced locomotor stimulation in mice

**Figure 2:**
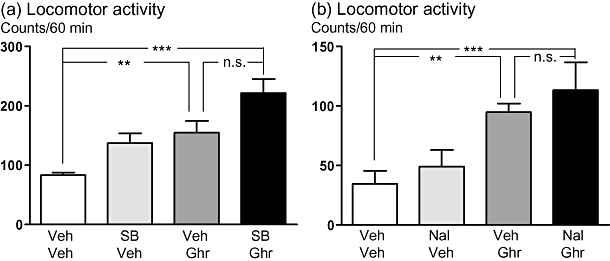
The ghrelin-induced locomotor stimulation is not affected by an orexin A antagonist (SB334867) or an opiod receptor antagonist (naltrexone). (a) Ghrelin (0.33 mg/kg)-induced locomotor stimulation was not affected by VTA administration of SB334867 (5 µg/side) in mice (F(3,24) = 8.44, *P* = 0.005) (*n* = 6–8; **P < 0.01, ***P < 0.001 and n.s. *P* > 0.05, Tukey's HSD post-hoc test). (b) Ghrelin (0.33 mg/kg)-induced locomotor stimulation was not affected by peripheral administration of naltrexone (1 mg/kg) in mice (F(3,28) = 6.01, *P* = 0.003) (*n* = 8; **P < 0.01, ***P < 0.001 and n.s. *P* > 0.05, Tukey's HSD post-hoc test).

The ghrelin-induced locomotor stimulation (*P* < 0.01) was not affected by VTA administration of the orexin A receptor antagonist SB334867 (*P* > 0.05) in mice (F(3,24) = 8.44, *P* = 0.005: *n* = 6–8) (Fig. 2a). Likewise, the ghrelin-induced locomotor stimulation (*P* < 0.01) was not suppressed by i.p. injection of the opioid receptor antagonist naltrexone (*P* > 0.05) in mice (F(3,28) = 6.01, *P* = 0.003: *n* = 8) (Fig. 2b).

### Effects of intra-VTA administration of a NMDA receptor antagonist on ghrelin-induced locomotor stimulation and increased accumbal dopamine release in mice

**Figure 3:**
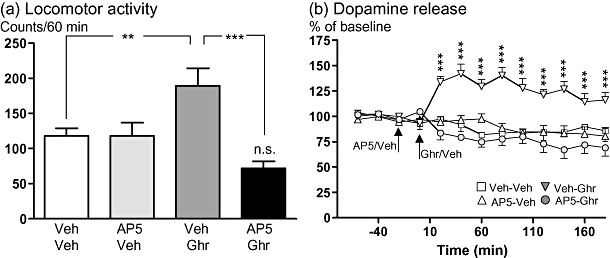
The ghrelin-induced locomotor stimulation and increased accumbal dopamine release are abolished by VTA treatment of the NMDA receptor antagonist AP5. (a) Ghrelin (0.33 mg/kg)-induced locomotor stimulation was attenuated by VTA administration of AP5 (0.5 µg/side) to but not by vehicle injection in mice (F(3,27) = 8.06, *P* < 0.001) (*n* = 7–8; **P < 0.01, ***P < 0.001; n.s. *P* > 0.05 for Veh-Veh vs. AP5-Ghrelin, Tukey's HSD post-hoc test). (b) We first demonstrated a significant effect of systemic ghrelin to increase dopamine release in comparison to vehicle treatment (*P* < 0.001) and secondly we showed that pre-treatment with AP5 (into the VTA) attenuated the ghrelin-induced increase in dopamine release compared to vehicle pre-treatment (*P* < 0.001) (treatment F(3,36) = 19.98, *P* < 0.001; time F(12,432) = 3.46, *P* < 0.001; treatment-time interaction F(12,432) = 5.49, *P* < 0.001). This difference was evident at the time intervals 20–180 minutes (*n* = 8–11; ***P < 0.001, Tukey's HSD post-hoc test).

Intra-VTA administration of the NMDA receptor antagonist, AP5, abolished the ghrelin-induced locomotor stimulation and accumbal dopamine release (Figs 3a,b), at a dose that had no effect *per se* (Table 1). Specifically, the ghrelin-induced locomotor stimulation (*P* < 0.01) was attenuated by VTA administration of AP5 (*P* < 0.001) in mice (F(3,27) = 8.06, *P* < 0.001: *n* = 7-8). Moreover, systemic ghrelin increased dopamine release (*P* < 0.001) and pre-treatment with AP5 attenuated the ghrelin-induced increase in dopamine release compared to vehicle pre-treatment (*P* < 0.001) (treatment F(3,36) = 19.98, *P* < 0.001; time F(12,432) = 3.46, *P* < 0.001; treatment-time interaction F(12,432) = 5.49, *P* < 0.001). This difference was evident at the time intervals 20–180 minutes (*P* < 0.001: *n* = 8–11).

**Table 1:** Effects of intra-VTA administration of different doses of the NMDA receptor antagonist, AP5, on locomotor activity in mice per se

*Dose AP5 (µg/side)*	*Locomotor activity (counts/60 minutes)*
0	981 ± 117
0.5	901 ± 43 n.s.
1	449 ± 192**
2	104 ± 25***

The NMDA receptor antagonist, AP5, decreased the locomotor activity at a dose of 1 µg/side and of 2 µg/side bilaterally into the VTA *per se* compared to vehicle treatment in mice. However, a dose of 0.5 µg/side bilaterally into the VTA did not affect the locomotor activity *per se* (F(3,12) = 12.72, *P* = 0.0005, *n* = 4 in each group.

** *P* < 0.01,

*** *P* < 0.001, n.s. *P* > 0.05, Tukey's HSD post-hoc test).

Control experiments showed that neither VTA administration, the volume infused nor the antagonist *per se* had any effect on locomotor activity (Figs 1a, 2a,b and 3a) or accumbal dopamine release (Figs 1b and 3b).

### Probe and cannula/e placements

**Figure 4:**
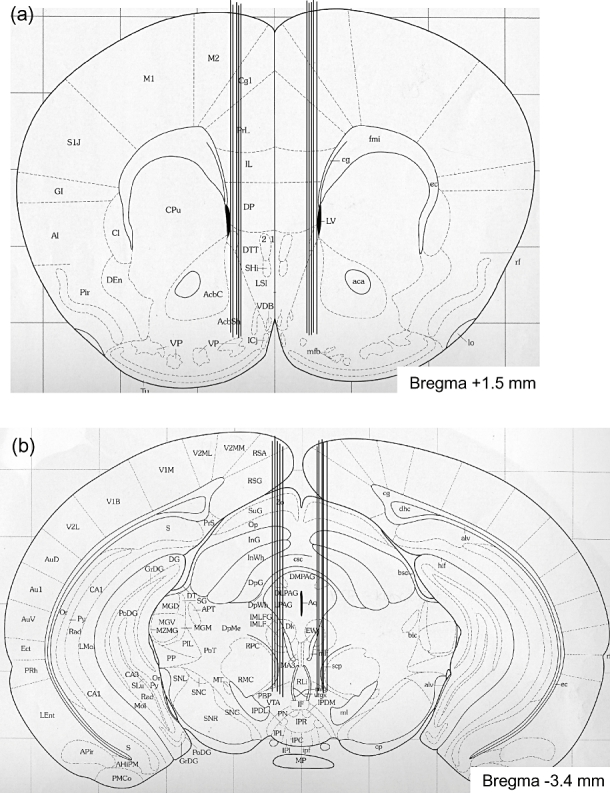
Verification of cannula/e and/or probe placement. A coronal mouse brain section showing ten representative probe placements (illustrated by vertical lines) in the N.Acc. (a) or guide cannula/e placements in the VTA (b) of mice used in the present study (Franklin & Paxinos 1996). Ten representative placements are illustrated, but all other placements were within the N.Acc. shell or in the VTA. Placements outside either of these areas were not included in the statistical analysis. The number given in each brain section indicates millimetres anterior (+) and posterior (−) from bregma.

For all locomotor activity experiments eight mice in each treatment group were implanted with bilateral guide cannulae, whereas 11 mice undertook surgery for each treatment group for the microdialyis experiments. All these mice were included in the experiments (locomotor activity or microdialysis studies). After the experiment the location of the probe and/or guide cannulae was verified and only mice with probe placement in the N.Acc. shell and/or cannula/e in the VTA were included in the statistical analysis (Fig. 4). Neither AP5 nor BIM28163 suppressed ghrelin-induced locomotor stimulation or accumbal dopamine release in a few mice in which the canulae were misplaced in neighbouring structures. It should also be emphasized that in a few mice the probe was located outside the N.Acc. shell and in these mice no effect of ghrelin on accumbal dopamine release was observed (data not shown).

## DISCUSSION

The present study demonstrates that the stimulatory effects of peripheral ghrelin on locomotor stimulation and accumbal dopamine release involve VTA GHS-R1A signalling as both effects were suppressed by VTA administration of a GHS-R1A antagonist (BIM28163). Moreover, ghrelin's ability to activate the mesolimbic dopamine system was suppressed by pharmacological blockade of glutamatergic receptors but not by blockade of opioid or orexin A receptors. Thus, the locomotor stimulating effect of ghrelin was not affected by intra-VTA administration of the orexin A antagonist (SB334867) or by peripheral administration of an opioid receptor antagonist (naltrexone). Finally, ghrelin-induced locomotor stimulation as well as accumbal dopamine release were suppressed by VTA administration of an NMDA receptor antagonist (AP5). Taken together these data suggest that systemic ghrelin activates the mesolimbic dopamine system via GHS-R1A in the VTA and that glutamate rather than orexin or opioid signalling is required for ghrelin to stimulate the mesolimbic dopamine system.

Given that transport of ghrelin across the blood-brain barrier into the brain is somewhat limited (Banks *et al*. 2002), there have been suggestions that ghrelin may exert its central effects via vagal afferents (Date *et al*. 2000) or by gaining access at circumventricular organs, such as the arcuate nucleus and area postrema. Studies using Fos protein to map ghrelin's central actions have shown that whereas peripheral administration of ghrelin and GHS-R1A agonists activate a rather limited population of cells in the arcuate nucleus (Dickson, Leng & Robinson 1993; Hewson & Dickson 2000) and area postrema (Bailey *et al*. 2000), additional hypothalamic cell groups were recruited after central administration (Lawrence *et al*. 2002). In the present study, it was demonstrated that the actions of peripheral ghrelin on the midbrain dopamine system, reflected by locomotor stimulation and accumbal dopamine release, can be blocked by VTA administration of a GHS-R1A antagonist. Taken together with previous neuroanatomical and accumbal dopamine measurement studies showing that the target cells for ghrelin in the VTA include the dopaminergic cell group (Abizaid *et al*. 2006; Jerlhag *et al*. 2006; Jerlhag 2008; Kawahara *et al*. 2009; Quarta *et al*. 2009), it seems likely that peripheral ghrelin directly activates the VTA dopamine system via GHS-R1A. The activation of the mesolimbic dopamine system by ghrelin may be due to the reported ability of the GHS-R1A to dimerize with the dopamine D1 receptor, both receptors expressed on dopamine neurons in the VTA, and thereby amplifies the dopamine signalling (Jiang, Betancourt & Smith 2006). Central ghrelin signalling system, including the GHS-R1A, appears to be required for reinforcement induced by addictive drugs including cocaine (Wellman, Davis & Nation 2005; Davis, Wellman & Clifford 2007; Tessari *et al*. 2007) and alcohol (Jerlhag *et al*. 2009). The mesolimbic dopamine system appears to be a likely target for ghrelin also in man, as evidenced from functional MRI studies in which peripheral ghrelin altered the response of the ventral striatum to visual food cues (Malik *et al*. 2008). Indeed, ghrelin may, via VTA GHS-R1A, increase the incentive value for natural as well as chemical reinforcements.

Neurotransmitters, including orexin, opioids as well as glutamate, have previously been shown to modulate the intake of natural and chemical reinforcers of the mesolimbic dopamine system as well as to regulate the activity of these VTA neurons (Hoebel *et al*. 1989; Engel *et al*. 1992; Wise 2002; Thiele *et al*. 2003). Specifically, AP5 and SB334867, in a similar dose range, attenuates the reinforcing properties of drugs of abuse (Herz 1997; Taber & Fibiger 1997; Smith *et al*. 2009). Here, neither the opioid receptor—nor the orexin A receptor—antagonist affected ghrelin-induced locomotor stimulation, indicating that these systems do not interfere with these effects of ghrelin. Consistent with our findings, the orexigenic response to ghrelin when administered into key mesolimbic dopamine structures such as the VTA has previously been shown to be independent of opioid receptor signalling (Naleid *et al*. 2005). Peripheral injection of naltrexone has been shown to blocks the reinforcing properties of alcohol in rodents (Herz 1997), suggesting that naltrexone passes the blood-brain barrier and has central effects. Even though opioid receptors, specifically the µ receptor, mediate drug-induced reinforcement this receptor appear to be less important for the ability of ghrelin to activate the mesolimbic dopamine system. Orexin-containing neurons have previously been suggested to regulate ghrelin-induced feeding (Toshinai *et al*. 2003), but orexin A receptors do not appear to be crucial for the locomotor stimulatory effects of ghrelin. Collectively, these data suggest that ghrelin-induced activation of the mesolimbic dopamine system appears to be regulated via other mechanisms than its orexigenic properties. In the present study, it was shown that ventral tegmental NMDA receptors are required for ghrelin-induced locomotor stimulation and accumbal dopamine release. Supportively, the effects of ghrelin to increase the electrical activity of dopaminergic neurons in the VTA appears to be dependent on the excitatory glutamatergic input and also blockade of NMDA receptors in the VTA reduces food-induced accumbal dopamine release (Taber & Fibiger 1997; Abizaid *et al*. 2006). NMDA receptors have also been shown to mediate the accumbal dopamine release observed when animals consume food after ghrelin administration (Kawahara *et al*. 2009). As shown previously, ghrelin-induced reinforcement also involves nicotinic acetylcholine receptors in the VTA (Jerlhag *et al*. 2006; Jerlhag *et al*. 2008). Collectively, these data suggest that neurotransmitters including acetylcholine and glutamate are required for ghrelin-induced reinforcement, which it has in common with natural as well as chemical reinforcers of the mesolimbic dopamine system. The mechanisms for the interaction between ghrelin, acetylcholine and glutamate in the VTA are still unclear. However, presynaptic nicotinic acetylcholine receptors have been shown to modulate the release of glutamate in the VTA, which via postsynaptic NMDA receptors regulate accumbal dopamine release (Schilström *et al*. 1998; Schilström *et al*. 2000). Tentatively, ghrelin may increase the acetylcholine release, which via such mechanisms may indirectly activate the mesolimbic dopamine system. However, the possibility of the existence of presynaptic NMDA receptors on cholinergic neurons cannot be excluded (Corlew *et al*. 2008). The possibility that ghrelin has an ability to rearrange the excitatory NMDA-mediated synaptic input in a manner that would increase the probability of activation of the dopamine neurons by other inputs should also be considered; thus findings in the hippocampus as well as VTA show that ghrelin has effects on synaptic plasticity (Abizaid *et al*. 2006; Diano *et al*. 2006). The glutamatergic afferents to the VTA mainly originate from the prefrontal cortex, lateral hypothalamus, bed nucleus of stria terminalis, superior coliculus and the LDTg. This input regulates the activity of dopamine via NMDA receptors (Schilström *et al*. 1998; Georges & Aston-Jones 2002; Geisler & Zahm 2005). It should however be emphasized that other afferents including GABA, serotonin and noradrenalin, known to modulate the activity of dopamine projections to the N.Acc. (Wise 2002), may also have important roles for the ghrelin-induced activation of the mesolimbic dopamine system. Although, GABA_A_ receptors in the VTA does not mediate the increase in accumbal dopamine observed after food consumption induced by ghrelin (Kawahara *et al*. 2009).

In summary, the present study shows that the effects of peripheral ghrelin on locomotor stimulation and accumbal dopamine release in mice (that reflect direct actions of ghrelin at the level of the mesolimbic dopamine system, specifically the VTA) can be suppressed by an NMDA antagonist and are therefore likely to be under glutamatergic control. These data may have clinical implications since hyperghrelinemia is associated with addictive behaviours including compulsive overeating and alcohol use disorder (Cummings *et al*. 2001; Kim *et al*. 2005; Kraus *et al*. 2005). It may therefore be proposed that ghrelin-responsive circuits at the level of the VTA, that appear to be sensitive to cholinergic and glutamatergic input, may serve as a novel pharmacological target for treatment of such addictive behaviours.
